# Takotsubo Cardiomyopathy: Current Treatment

**DOI:** 10.3390/jcm10153440

**Published:** 2021-08-02

**Authors:** John E. Madias

**Affiliations:** 1 Icahn School of Medicine at Mount Sinai, New York, NY 10029, USA; madiasj@nychhc.org; Tel.: +1-(718)-334-5005; Fax: +1-(718)-334-5990; 2 Division of Cardiology, Elmhurst Hospital Center, Elmhurst, NY 11373, USA

**Keywords:** takotsubo syndrome, takotsubo cardiomyopathy, therapy of takotsubo syndrome, therapy of takotsubo cardiomyopathy

## Abstract

Management of takotsubo syndrome (TTS) is currently empirical and supportive, via extrapolation of therapeutic principles worked out for other cardiovascular pathologies. Although it has been emphasized that such non-specific therapies for TTS are consequent to its still elusive pathophysiology, one wonders whether it does not necessarily follow that the absence of knowledge of TTS’ pathophysiological underpinnings should prevent us for searching, designing, or even finding, therapies efficacious for its management. Additionally, it is conceivable that therapy for TTS may be in response to pathophysiological/pathoanatomic/pathohistological consequences (e.g., “myocardial stunning/reperfusion injury”), common to both TTS and coronary artery disease, or other cardiovascular disorders). The present review outlines the whole range of management principles of TTS during its acute phase and at follow-up, including considerations pertaining to the recurrence of TTS, and commences with the idea that occasionally management of TTS should consist of mere observation along the “first do no harm” principle, while self-healing is under way. Finally, some new therapeutic hypotheses (i.e., large doses of insulin infusions in association with the employment of intravenous short- and ultrashort-acting β-blockers) are being entertained, based on previous extensive animal work and limited application in patients with neurogenic cardiomyopathy and TTS.

## 1. Introduction

This review focuses exclusively on the treatment of patients with the acute phase of takotsubo syndrome (TTS), the follow-up management of this malady, and its recurrence. A disclaimer is in order in the outset regarding the term “takotsubo cardiomyopathy” since TTS is a syndrome, and not a cardiomyopathy [1], although the use of the term “takotsubo cardiomyopathy” may be excused herein, since the present review is published as part of a Special Issue of the *journal*, “Cardiomyopathies: Current Treatment and Future Options”. Reference to symptoms, signs, laboratory testing, diagnostic imaging, complications, and pathophysiologic and prognostic considerations are cursorily mentioned and discussed, merely as they pertain to the different treatments of TTS, the underlying justification for their employment, the patients’ response to such treatments, and side-effects arising thereof.

The pathophysiology of TTS continues to elude us [2,3,4]; however, it appears that an autonomic sympathetic nervous system (ASNS) seethe with resultant intense stimulation of cardiomyocytes via norepinephrine [5] and/or the cardiomyocytes’ damaging effects of blood-borne catecholamines (mainly epinephrine), secreted by the adrenals [6], are instrumental to the TTS pathophenotype, a condition thus conceptualized as a “chemical myocarditis”. Other plausible pathophysiologic scenarios include epicardial coronary artery spasm involving many vessels or coronary branches (i.e., some form of relatively prolonged Prinzmetal’s angina) [7], coronary microvascular spasm, endothelial dysfunction, or some phenotype of coronary artery disease (CAD), any combination of the above, or in association with other mechanisms, leading to stunning/reperfusion myocardial injury, not unlike the one encountered in CAD-related ischemic injury (acute coronary syndromes [ACS], or acute myocardial infarction [AMI]) [4]. According to the latter pathophysiologic pathway, one should view with tolerance and consider the testing and/or employment of therapies designed for CAD/ACS/AMI, in the management of acute TTS [8,9,10,11]. 

## 2. Therapies as Related to the Pathophysiology of Acute TTS

It has become a cliché that we lack a specific therapy for TTS, because we have not secured a definitive pathophysiologic etiology of TTS. This may partially be a plausible assertion. If the pathophysiologic underpinnings of TTS (e.g., intense ASNS-derived cardiostimulation) which have triggered the disease are operating to the same degree, or most probably at a decreased intensity [5,6], after the patients with TTS come under our care, therapies bridling autonomic sympathetic hyperactivity may be considered appropriate (e.g., employment of β-blockers in the acute and subacute phases of TTS). Additionally, it is conceivable that, if TTS, pathophysiologically speaking, is linked to coronary vasospasm [1,2,3,7], nitroglycerine, organic nitrates, or calcium-blockers may be considered as appropriate therapies. Finally, if TTS is considered as a subtype of CAD or AMI [4], with underlying pathohistological features of “stunned myocardium/reperfusion injury”, therapies designed for AMI, should be considered management approaches deserving evaluation [8,9,10,11]. 

While we are talking about our quest for unravelling the pathophysiology of, and thus providing specific therapies for TTS, we should be cognizant of the sobering fact that patients admitted with various clinical syndromes eventually diagnosed as TTS, are cared for, over many hours to sometimes days, with the provisional diagnosis(es) of CAD, AMI, or other cardiovascular or non-cardiovascular nature [12], until and even after the diagnosis of TTS has been established, following coronary angiography, showing normal coronaries or non-obstructive CAD. Accordingly, it is expected that such patients receive non-specific or empirical therapies, for many hours to days until coronary angiography discloses the absence of CAD and coronary thrombus, or the presence of non-obstructive CAD. Consequently, it may be inevitable, and not inappropriate, to treat patients with acute TTS, employing therapies for CAD, AMI, and other cardiovascular syndromes. Indeed, the commonality excessively voiced that we need special, evidence-based medicine TTS-directed randomized controlled trials (RCT) to decide on specific therapies for TTS may be impractical and perhaps not even necessary, because even at the time point of patients’ hospital admission, TTS has probably been “finalized”, and the condition is in its process of recovery. The above constitutes a personal opinion expressed repeatedly [6,11], may apply only to a subset of patients with TTS, and can be tangentially supported by the fact that the diagnosis of TTS is often made with considerable delay, after patients have been treated sometimes for several days as having ACS or AMI, by the rapid partial or complete recovery of the left ventricular (LV) function (sometimes within hours to 2 days), following prompt clinical presentation after the onset of the illness [13,14,15,16], or by the fact that some patients with TTS are found to have normal cardiac troponin values and/or almost normal LV function shortly after admission. Thus, therapy needs to focus on the management of established TTS pathophysiologic/pathologoanatomic/pathohistologic consequences, and complications.

## 3. Current Therapy of Acute TTS

**Figure 1:**
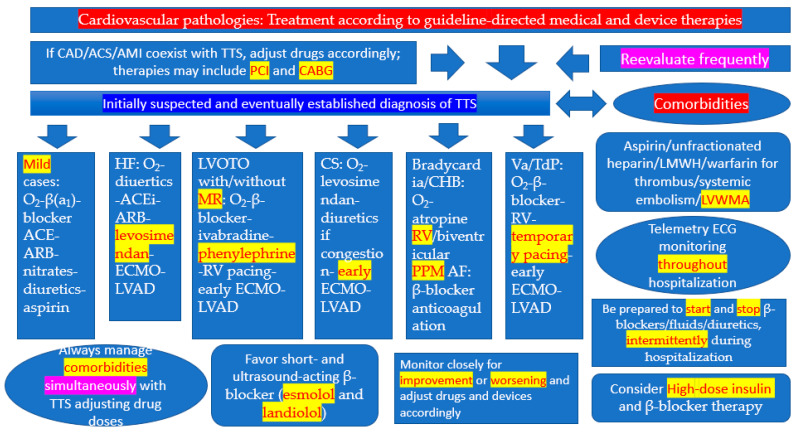
A graphic decisional tree of the bedside conceptualization of therapy for patients with TTS.

What follows is a distillation of therapies practiced/proposed in the 5,534 papers, as of 31 July 2021, accessed in PubMed in response to the MeSH term “takotsubo” [17]. Many papers on TTS contain some information pertaining to its therapy [18], while some publications are focused exclusively on the management of TTS [19]. Recommendations herein are provided with the proviso that the diagnosis of TTS has been established and coronary angiography has excluded obstructive CAD, ACS, or AMI. *It may be advisable for the reader to peruse and contemplate the contents of Table 1 and Figure 1, before continuing reading of the following sections discussing individual complications.*

### 3.1. Asymptomatic/Normotensive/Normocardic Patients with TTS

When patients with TTS are asymptomatic with normal blood pressure (BP) and heart rate (HR), and their chest pain, dyspnea, or other symptoms or signs of disease, which brought them to seek medical attention have abated, supportive care suffices, along the lines of the Hippocratic “primum non nocere” (“first do no harm”) principle [20,21,22]. Indeed, one should be cognizant of the possibility that pharmacological interventions in mild cases of TTS may contribute to complications, otherwise not expected had the natural course was left to evolve without any iatrogenic interference [21]. Certain drugs taken for previously present comorbidities should not be held. A short course of limited anticoagulation therapy may be needed to prevent stroke, systemic embolism, or pulmonary embolism [20,21,22,23], particularly if there is sizeable apical/midventricular akinesis/dyskinesis with apical ballooning, which predisposes to thrombus formation when coupled with the sympathetic overdrive, which induces hypercoagulability [24,25], even in asymptomatic patients and in the absence of heart failure (HF), while the patient is self-healing. In reference to prophylactic administration of anticoagulation, restraint should be exercised until one has excluded the presence of ACS or AMI via coronary angiography [21]; in the same vein, one should avoid using anticoagulants if it is suspected or shown that the underlying trigger for the TTS episode was intracerebral bleeding [21,26,27,28]. Continuous electrocardiographic (ECG) monitoring for emergence of arrhythmias and for QTc prolongation [29], associated with ventricular arrhythmias (VA), should be instituted and maintained throughout hospitalization, and even beyond, if left ventricular (LV) wall motion abnormalities (LVWMA), or LV thrombus, detected during hospitalization, persist at follow-up [20,30,31,32,33]. Although some physicians continue or start angiotensin-converting enzyme inhibitors/angiotensin receptor blockers (ACEi/ARB), β-blockers, diuretics and aspirin (in patients with a history of atherosclerosis or CAD), one should not forget that any therapy not proven in patients with TTS, particularly when they are asymptomatic, should be considered “quasi-experimental”, tentative, and subject to close monitoring (*which applies to ALL pharmacological or other therapies administered*), continuation, or termination, depending on the response of the patients. Any treatment recommendations discussed herein emanate from general clinical reasoning consensus among experts, observational studies, and case series of patients with TTS (level of evidence C) [21,22]. Intuitively, inclusion of β-blockers may be justified, considering the nosogenic role of catecholamines in TTS; however, patients have recovered without the use of such therapy, and there is no evidence that the catecholamine-based injurious effect continues to be exerted hours or days after the inception of disease, when patients come under our care, and thus therapy with β-blockers is essential. On the other hand, precipitation of TTS by withdrawal of metoprolol in a patient has occurred [34], although that patient had LV outflow tract obstruction (LVOTO), where the β-blocker was indicated. Various cardioselective β_1_-blockers (e.g., metoprolol [35], bisoprolol, esmolol [short-acting] [21,36], landiolol [ultrashort-acting]) [37,38,39], non-cardioselective (propranolol) [40,41] or non-cardioselective β-blockers with associated α_1_-blocking effects (e.g., carvedilol or labetalol [40] have been used in patients with TTS, but no head-to-head comparisons of these drugs have been undertaken. There is also literature supporting the view that β-blockers are not beneficial in patients with TTS [42], as also shown by the reports revealing that a sizeable proportion of patients on a maintenance therapy with β-blockers have suffered TTS [43]. In general, and in reference to the employment of ACEi/ARB), β-blockers, calcium channel blockers, and aspirin, based on the literature, summarized elsewhere [20], there is no support to initiate them in asymptomatic or mildly symptomatic patients with TTS, extrapolating from the established HF management norms. Even when the above drugs are initiated on admission because of mild symptoms, there should be close monitoring to evaluate whether such therapies have not resulted in worsening in the patients. There is no other substitute than the close hemodynamic assessment of patients with TTS, particularly in the early phase of the disease (Figure 1). 

The mindset in implementing therapies in patients with TTS should include the notion that the TTS phenotype may be an evolution-based protective biological algorithm to prevent death, and thus the caring physician should exercise restraint for a reflex-like implementation of pharmacology by extrapolating therapeutic modes employed in other cardiovascular pathologies [20]. Indeed, the emphasis should be on supportive care to avoid complications, while the self-restorative process to normalcy is under way. 

### 3.2. Angina in Patients with TTS

Angina should be managed with sublingual or intravenous nitroglycerin, organic nitrates, with care not to precipitate intensification of mid-LV gradient, mediated by a reduction in systemic vascular resistance (SVR), in patients with complicated LVOTO [20]; β-blockers can also be given for angina, which may also help in alleviating LVOTO [20,35,36].

### 3.3. Dyspnea in Patients with TTS 

Patients with TTS presenting with dyspnea should be monitored closely, regarding their hemodynamic changes and blood oxygen saturation, and insight about the extent of LV and right ventricular (RV) dysfunction, and degree of lung congestion should be sought promptly by auscultation, chest X-ray, transthoracic echocardiography (ECHO), and lung ultrasound. Diuretics, nitrates, and β-blockers, depending on the presence/absence of tachycardia, hypertension, bradycardia, hypotension, and evidence of LVOTO, may suffice. Mechanical respiratory support may be needed when pulmonary edema ensues with no response to drugs [22], and patients should be monitored for abrupt decompensation and the need for implementation of mechanical circulatory support (MCS) [22] (Section 3.13).

### 3.4. Hypertension and/or Tachycardia in Patients with TTS

β-blockers can be given for high BP and/or HR; short- or ultrashort-acting β-blockers should have preference, particularly during the early course of TTS, followed later by metoprolol or bisoprolol [36]. Additionally, ivabradine has been used to ameliorate sinus tachycardia [44]. 

### 3.5. Hypotension in Patients with TTS

Often patients with TTS are hypotensive on admission to the hospital, but the mechanism of this phenomenon may be an underlying decreased SVR, one of the hallmarks of TTS [20,21], mediated by downward perturbation of the sympathetic activity [45], and/or an enhanced parasympathetic activity [46], occasionally encountered in patients with TTS [5]. This can be managed by an increase in the intravenous fluid intake. Low BP should be “tolerated” providing that the cardiac output is adequate, or the organ perfusion is well maintained; however, patients receiving enhanced fluid infusions should be monitored closely (lung auscultation, blood oxygen saturation, mental state, urine volume, and the patients’ subjective feeling of well-being) for adequacy of organ perfusion or emergence of pulmonary congestion. With persisting hypotension, β-blockers should not be administered, or if they have been started, they should be discontinued [19], and following adequate fluid administration, phenylephrine, an α_1_-agonist should be considered in preference to positive inotropic drugs (e.g., norepinephrine) [20,21]. Additionally, continuous vigilance for present or emerging LVOTO and mitral regurgitation (MR), should be exercised via frequent application of ECHO [21].

### 3.6. Bradycardia and/or Atrioventricular Blocks in Patients with TTS

**Figure 2:**
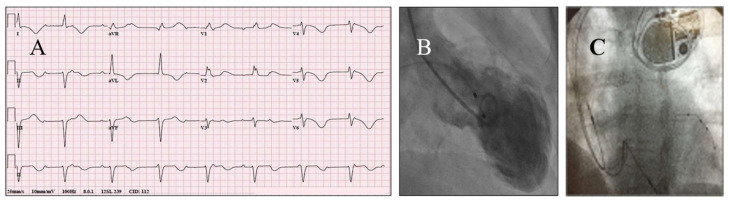
A patient with TTS who received a PPM for persisting CHB. A 62-year-old woman suffered TTS associated with CHB and inverted T-waves in the lateral ECG leads (**A**), for which she was initially transvenously paced, and subsequently received a PPM (biventricular) (**C**), because her CHB was persistent; ventriculography showed apical ballooning with a LVEF of 35% (**B**); an echocardiogram 15 days after the admission revealed a LVEF of 50%. CHB occasionally represents a complication of TTS, while in some cases it is the precipitant of TTS. Often, the CHB persists after the time point of normalization of LV function, and in such circumstances, there is a need for a PPM implantation. When the CHB resolves before the normalization of the LV function, a PPM may not be necessary. The CHB may be of the supra-Hisian (narrow QRS complexes) like in the present case, or of the intra-Hisian (wide QRS complexes) variety, and could precipitate VA, including TdP, mediated by the bradycardia resulting from the CHB. The present patient had a car accident due to her CHB, showed a prolonged QTc, and developed a brief episode of TdP; she was discharged with a PPM and a life vest, with an ICD not required. Reproduced and modified from Ref. [49], with the permission of the Baylor University, Medical Center.

Sinus bradycardia or asystole are occasionally seen in patients with TTS [5], and can be managed with successive small doses of atropine. β-blockers or other drugs causing bradycardia should not be administered, or if they have been started, they should be promptly discontinued [19]. Atropine should also be used in patients with mild atrioventricular (AV) blocks, but advanced or complete heart block (CHB) should be managed with a temporary pacemaker, to be followed by permanent pacemaker (PPM) implantation decided on an individual basis [22,47,48,49] (Figure 2). Not all patients with TTS and CHB require PPM implantation, but some patients who have suffered TTS, triggered by CHB, certainly need to have a PPM implanted without exception [47,50]. The persistence of CHB at follow-up in patients with TTS indicates not only the need for PPM in many patients with TTS presenting or developing this complication during the acute phase of the illness, but that TTS itself most probably had been triggered by an underlying previously present AV conduction abnormality [22,48]. Remarkably, patients receiving PPM have not required further therapy for malignant VA at follow-up [51]. 

### 3.7. LVEF < 30% and/or Large Apical Akinesis/Dyskinesis in Patients with TTS

Patients with a LVEF of <30% and/or marked apical akinesis/dyskinesis have worse in-hospital and follow-up prognosis [21,22], and should be closely monitored for emergence of HF, cardiogenic shock (CS), atrial arrhythmias and lethal VA, and thrombus formation [22,23]. Due to the latter, the threshold for prophylactic anticoagulation should be lower, until the LV function is markedly improved or restored to normal (Section 3.8) [21,22]. 

### 3.8. Thrombus and Prevention of Embolism in Patients with TTS

**Figure 3:**
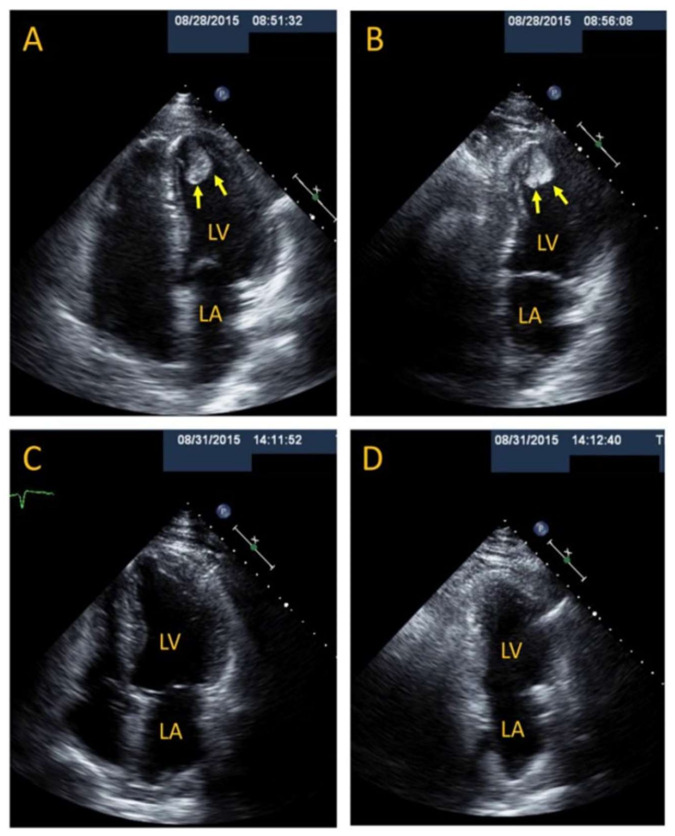
A patient with TTS and LV thrombus resulting in systemic embolism. A 53-year-old man suffered TTS following intense emotional stress and underwent thrombectomy for acute right superficial femoral and popliteal arterial thrombosis. Cardiac exam and troponin values were normal, and ECG showed non-specific ST-T wave changes. ECHO (4-chamber (**A**) and 2-chamber (**B**)) revealed a LV apical pedunculated and mobile thrombus measuring 2.4 × 2 cm, LV apical ballooning, and LVEF of 40% to 45%. Luminal irregularities were found at coronary angiography. A repeat ECHO 3 days after starting unfractionated heparin showed complete resolution of LV thrombus and apical akinesis (**C**,**D**). He was discharged in stable condition on warfarin for at least 3 months. LV thrombus with or without systemic embolism in TTS can occur early or late in the clinical course, and with rapid or delayed resolution. Immediate initiation of anticoagulation is warranted, and a repeat ECHO within days to 1 week is advisable to follow its course and possible early resolution. cCTS and cMRI can provide additional information. Duration of anticoagulation can be decided upon in consideration with a patient’s benefit–risk ratio. Reproduced and modified from Ref. [52], with the permission of the Journal of Investigative Medicine High Impact Case Reports.

Thrombus occurs particularly in patients with a low LVEF (i.e., <30%) [21] and marked apical ballooning, and it may appear very early in the clinical course or late, 2 weeks after the inception of the illness [22,52] (Figure 3). Management of LV and/or RV, left atrial, and even left atrial appendage [53] thrombus via anticoagulation is discussed also in Section 3.1; prevention is based on early and frequent use of ECHO to detect very early development of thrombus sometimes on admission or just a few hours thereafter, or development of severe LVWMAs [20,21,23,52,54]. Unfractionated heparin, low-molecular-weight-heparin (LMWH), vitamin K antagonists, aspirin, and/or P2Y12 receptor antagonists such as clopidogrel, prasugrel, or ticagrelor, or the new oral anticoagulants can be used [20,52,54], as in other cardiovascular pathologies. Because antiplatelet agents are among the drugs which according to some “should be a part of standard treatment and initiated early” [20], this does not imply that antiplatelet drugs in isolation suffice for the management of thrombus, or severe LV dysfunction and apical ballooning, which could predispose patients with TTS to thrombus development. The bulk of the literature supports the view that heparin should be initiated in the presence of thrombus, followed by warfarin until at follow-up the thrombus has resolved, with some adding aspirin to this antithrombotic regimen [55,56,57,58]. The issue of anticoagulation should be considered broadly, encompassing its consideration for patients with TTS and severe LVWMAs and their duration without presence of intraventricular thrombus, presence of thrombus, and concern about the role of anticoagulation in causing cardiac rupture, a rare complication of TTS [20,59]. Important issues are the vigilance for the emergence of thrombus, early implementation of anticoagulation, and its maintenance for 3 to 4 or 6 months, as the resolution of the thrombus and LV dysfunction takes place. 

### 3.9. Left Ventricular Outflow Tract Obstruction in Patients with TTS

**Figure 4:**
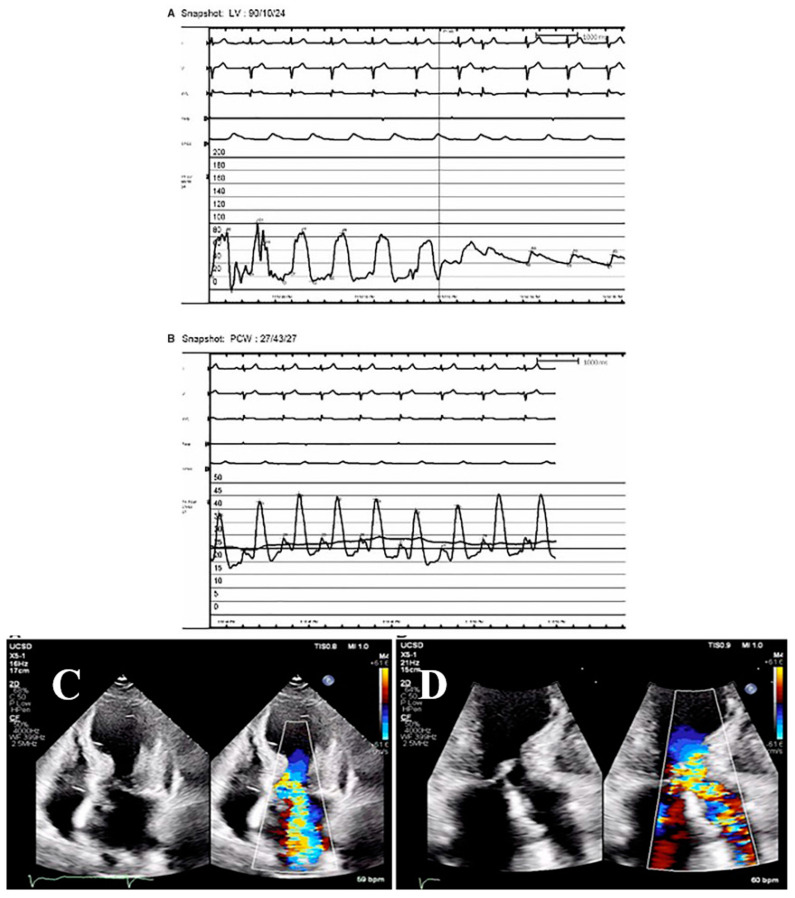
A patient with TTS, LVOTO, MR, and CS, responding to β-blocker and phenylephrine. A 71-year-old woman presented with chest pain, ECG lateral ST-segment elevations, and hypotension. Coronary angiography revealed no significant CAD, but a left ventriculogram showed TTS. RH catheterization revealed CS, elevated filling pressures, and V waves due to severe MR (**B**), while a dynamic LVOTO was found on LV to aorta pullback (**A**). ECHO 4-chamber view showed LVOTO and MR due to anterior mitral valve leaflet SAM (**C**), and 3-chamber view showed increase velocities across the LV outflow tract. (**D**). Worsening resulted from starting dopamine and IABP, but she improved with the initiation of phenylephrine and a low-dose β-blocker. Repeat ECHO in 3 weeks showed complete resolution of LVOTO, MR, apical akinesis, and MR. LVOTO and MR respond favorably to fluid administration to improve preload, β-blocker therapy to increase diastolic filling time, and vasopressors to raise afterload. Reproduced and modified from Ref. [64], with the permission of the European Heart Journal—Case Report.

Intravenous metoprolol, esmolol, or landiolol was beneficial in patients with TTS and LVOTO via an increase in the diastolic filling time by a decrease in HR, and a decrease in contractility [21,22,35,36,39]. Consideration along with the β-blockers should be given to the use of α_1_-agonist (e.g., phenylephrine) [20,21] in patients with TTS and LVOTO, in an effort to increase a possibly decreased SVR, and to increase the afterload and/or to alleviate the LVOTO. In reference to the postulated decreased SVR, mediated by an underlying altered peripheral sympathetic nerve activity in some patients with TTS [45], perhaps monitoring of sympathetic nerve input to the heart via conventional ECG electrodes [60] may evaluate for ASNS perturbations. If LVOTO is associated with CS, an intravenous infusion of short-acting β-blockers (esmolol) should be cautiously implemented, providing that there are no signs and/or symptoms of cardiac decompensation [22]. Indeed, β-blockers should be avoided in the setting of TTS with complicated LVOTO and CS [21]. Ivabradine, in place of β-blockers, has been recommended for amelioration of LVOTO, mediated by a slowing of HR [21,61]; this drug has the advantage over β-blockers that it does not negatively affect ventricular contractility. Additionally, diuretics and nitrates should be avoided since they result in intensification of LVOTO, mediated by a reduction in LV preload [21]. Instead, fluid administration to improve preload should be considered, although initiation of diuretics for associated hypoxic respiratory failure due to pulmonary edema may be required in some cases. Occasionally, LVOTO is associated with MR mediated by LVOTO-induced anterior leaflet of mitral valve systolic anterior motion (SAM) abnormality. In recalcitrant cases of LVOTO with unsatisfactory response to β-blockers and α_1_-agonists, RV apical electrical pacing can be considered, extrapolating the reasoning advanced for patients with LVOTO due to obstructive cardiomyopathy [20,21,62]. Extracorporeal membrane oxygenation (ECMO) and LV assist devices (LVAD) should be considered in the management of LVOTO, and implemented relatively early, and not as the last resort when hemodynamic status has deteriorated [20]. Intra-aortic balloon pump (IABP) is contraindicated in patients with LVOTO due to its induced drop of afterload, precipitating further intensification of the intraventricular pressure gradient [20,21,51,63,64] (Figure 4), and considering the unfavorable response of patients with AMI to this modality [64].

### 3.10. Heart Failure in Patients with TTS

Positive inotropic agents and vasodilators should be avoided and early, instead of a delayed “heroic” treatment with MCS using venoarterial ECMO or LVAD should be considered [20,65,66,67,68]. Although close monitoring of hemodynamic consequences of pharmacological and MCS can be assessed by frequent clinical evaluation, serial noninvasive or invasive assessment of cardiac output, stroke volume, and SVR may be required in certain cases. HF may emerge in patients with TTS in association with a LV ejection fraction (LVEF) <40%, physical stressors and age >70 years [46]. ACEi/ARB, β-blockers, and diuretics are often employed, as in the management of HF resulting from other causes; however, β-blockers should not be administered, or if they have been started, they should be discontinued [19], when HF emerges or worsens, particularly in association with organ hypoperfusion. Indeed, β-blockers may be considered later, as recovery of LV dysfunction is under way, or completed [19]. Consideration of administration of a calcium sensitizer levosimendan, a non-cathecholamine inotrope, in patients with low cardiac output and HF [22,69] should be balanced with concern about the vasodilating effects of this drug, with resultant drop of the BP [20,69]. Additionally, it is reasonable to continue or start aspirin for patients with TTS and associated CAD. 

### 3.11. Mitral Regurgitation in Patients with TTS

MR in patients with TTS is seen in association with the ballooning of midventricular and apical LV, causing leaflet tenting or tethering independent of LVOTO, or with LVOTO leading to anterior leaflet SAM abnormality. Its management should follow the principles of therapy for LVOTO (Section 3.9) and alleviation of MR with unloading vasodilators, as for other cardiovascular conditions, when MR is not associated with LVOTO. MR may be partially related to “tenting” of mitral leaflets, mechanistically associated with the LV apical-mediated and overall heart chamber enlargement, and thus may respond to all pharmacologic and LVAD-based measures [65].

### 3.12. Right Ventricular Involvement in Patients with TTS

RV involvement in TTS afflicts at least 1/3 of patients, is underestimated by conventional ECHO, and strain ECHO imaging is more sensitive in its detection; also, it is associated with worse in-hospital prognosis and at follow-up [70]. Such RV involvement should place physicians on the alert for other anticipated complications. Patients with RV involvement should be monitored for hypotension, RV failure, RV thrombus, and possible need for anticoagulation and fluid infusion-based resuscitation [21].

### 3.13. Cardiogenic Shock in Patients with TTS

**Figure 5:**
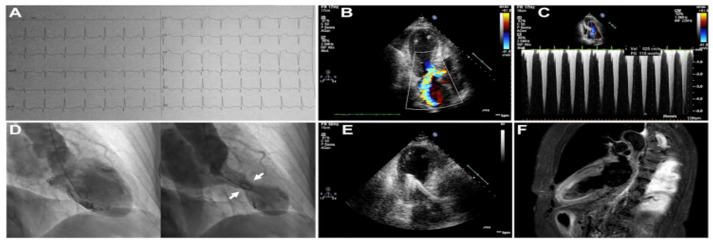
A patient with TTS, LVOTO, MR, and CS, treated with a percutaneous LVAD. A 71-year-old woman suffered TTS associated with CS, a LVEF of 30% (**B**,**D**), apical ballooning (**B**,**D**), LVOTO with a peak gradient of 110 mmHg (**C**), and severe MR due to anterior mitral valve leaflet SAM (**B**). An Impella 2.5 percutaneous ventricular assist device was implanted. Fluid was administered and intravenous esmolol at 50 μg/kg/min was infused. Within 4 h, LV function and lactate normalized. The LVAD was removed in 72 h, and the patient had complete recovery of her LVEF by day 30 of follow-up (**E**). The admission ECG showed ST-segment elevations in the anterolateral leads and q-waves in the inferior and anterolateral leads (**A**), and a cMRI revealed ME (**F**). MCS permits avoidance of inotropes, optimization of fluid administration, and employment of β-blockers. Reproduced and modified from Ref. [75], with the permission of the JACC: Cardiovascular Interventions.

Diagnosis of CS in patients with or without pulmonary congestion (sometimes escalated to frank pulmonary edema) and/or hypotension should be based on the presence of organ perfusion, since many patients may be hypotensive but not in CS [45]. Additionally, exclusion of LVOTO, MR, RV involvement, and cardiac rupture as the underlying mechanism of hypotension or systemic hypoperfusion and CS should be considered [21], and if found, managed accordingly (Section 3.9, Section 3.11, Section 3.12 and Section 3.14). Considering the postulated role of catecholamines in the pathophysiology of TTS and precipitation of LVOTO, inotropic drugs (e.g., adrenaline, noradrenaline, dopamine, dobutamine, milrinone, and isoprenaline) to counteract hypotension and/or CS are contraindicated. Pharmacological considerations should be implemented after estimating or even objectively measuring cardiac output or index, SVR, and organ perfusion [20]. Instead the employment of levosimendan, a novel calcium sensitizer, which exerts its inotropic effect by prolonging actin–myosin interaction, leaving adrenoceptors unaffected [20,69], is recommended for patients with TTS and HF and CS [22,71], although this matter needs further assessment [72]. An additional issue to be considered, pertaining to levosimendan, is its vasodilating effect, promoting hypotension, worsening LVOTO, and further lowering the occasionally present in patients with TTS low SVR [20,69]. Additionally, the phosphodiesterase inhibitor milrinone has been considered in patients with TTS and CS [69,72]. Others absolutely disfavor the use of inotropic drugs in patients with TTT and HF or CS [20,21,65,66,67,68], a view with which this author concurs. Indeed, it may be revealing to explore carefully whether the lower mortality noted in the SWEDEHEART TTS in comparison to the InterTAK TTS cohorts in general, and in men vs. women in particular, could be traced mainly to the lower use of inotropic drugs [73], providing one performs a careful propensity score matching of many important covariates and cofounders of the 2 registries. Persisting hemodynamic instability should be managed with MCS, implementing ECMO and/or LVAD (IABP, TandemHeart, and microaxial pumps (i.e., Impella™, Abiomed, Danvers, MA)) in a bridge-to-recovery management strategy [20,21,22,65,68,74,75]. (Figure 5). IABP initially advocated in the management of TTS complicating CS is not anymore favored [20,51], considering its equivocal role in AMI, and particularly in patients with TTS and LVOTO where it had precipitated further hemodynamic deterioration [20,21,51]. The use of MCS, although costly, has not led to improvement in mortality, and has been linked to an increased number of patients with TTS, discharged to skilled nursing facilities [65].

### 3.14. Heart Rupture in Patients with TTS

The devastating complication of cardiac rupture in patients with TTS may be prevented by the early employment of β-blockers [43]. Considering that the mean LVEF, systolic BP, and the double product, indicative of increased oxygen demands, were higher in patients who suffered cardiac rupture as compared to those without such complication, β-blockers may be indicated in patients with this phenotype [76]. Immediate surgical repair should be attempted [76], although there is literature of patient survival with TTS and ventricular rupture managed with conservative means [77]. Patients with LV rupture and conservative management, have revealed evidence of hemorrhagic pericardial effusion, tamponade, and LV thrombus, and have been managed with pericardial drainage, or pericardial window [77].

### 3.15. Atrial Arrhythmias in Patients with TTS

All types of atrial arrhythmias have been observed in patients with TTS. The commonest arrhythmia encountered is sinus tachycardia, which usually responds to β-blockers. It may be prudent to start with the use of intravenous esmolol or landiolol, since these drugs can be discontinued without impunity due to their short/ultrashort-action duration, in case their use has led to worsening of HF, CS, hypotension, bradycardia, or any other complications. Additionally, ivabradine has been recommended for alleviation of sinus tachycardia in patients with TTS [61]. Atrial fibrillation (AF) a common occurrence emerging in association with an episode of TTS [78] should be managed, primarily directed at anticoagulation (Section 3.8), and slowing of heart rate, initially based on intravenous short/ultrashort-acting β-blockers, and then transitioning to long-acting β-blockers. DC-cardioversion may be considered if necessary [21]. Correction of electrolyte abnormalities is paramount in the management of atrial arrhythmias [79]. Use of calcium channel blockers for the management of AF, digitalis (particularly in the presence of LVOTO [21]), or antiarrhythmic drugs (due to their association with VA, and since some of such drugs may precipitate prolongation of QTc [80], should not be considered during the hospitalization phase of TTS. 

### 3.16. Ventricular Arrhythmias in Patients with TTS

**Figure 6:**
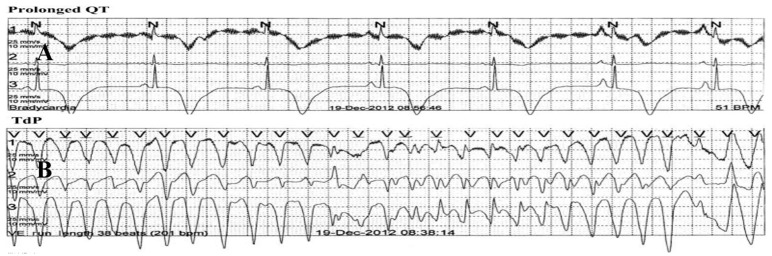

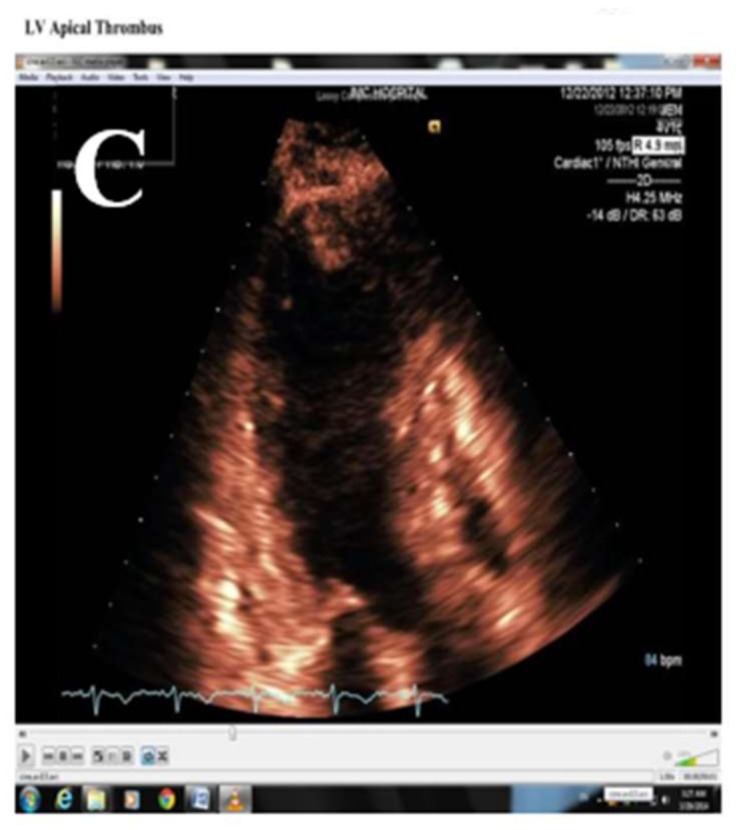
A patient with TTS, long QTc, and recurrent TdP and VF treated with an ICD. A 48-year-old woman with a history of postpartum depression, no CAD risk factors, and recurrent attacks of TTS of the apical variant phenotype, spanning 7 years (with the latest episode depicted herein), always precipitated by emotional stress, and resulting in chest pain, loss of consciousness, low LVEF, and sinus bradycardia (51 beats/min), inverted T-waves, long QTc (554 ms) (**A**), and repeated TdP episodes (**B**), and a LV thrombus (**C**). Coronary angiogram and LV function in between episodes were normal. She was DC-cardioverted for recurrent VF, had her β-blocker discontinued, and received magnesium, mexiletine, anticoagulation, and an ICD. LVEF normalized and the LV thrombus resolved. Reproduced and modified from Ref. [81] with the permission of the Journal of the Saudi Hear Association.

Patients with TTS experience VA (multiple premature ventricular contractions), ventricular tachycardias [VT] (both monomorphic and polymorphic [Torsades de Pointes] {TdP}), ventricular asystole, and pulseless electrical activity [81] (Figure 6). Vigilance for the presence of electrolyte disturbances is of paramount importance [21]. The association of VA with prolonged QTc is well established, and the need for continuous monitoring of the QTc during hospitalization has been strongly advocated [29,80]. Antiarrhythmic medications, antidepressants, or antibiotics associated with prolongation of the QTc should not be used, or promptly discontinued [22]. Treatment with β-blockers may also protect against malignant VA in patients with TTS [82], and in this setting, short-acting β-blockers should be favored [22]. Regarding the concern about the effect of β-blockers on the QTc, the issue was recently investigated, and reassuringly no prolongation of the QTc, which could have been attributed to these drugs, was noted during the first 3 days of hospitalization [83]. Sustained VA associated with CS may require MCS implementing ECMO and/or LVAD [21]. Hypokalemia should be corrected, drugs precipitating bradycardia should be avoided [19], and if such predisposing complications emerge, particularly when prolonged QTc is present, RV pacing for a protective mild increase in the underlying slow HR may be needed [78]. Episodes of TdP should be treated with DC-cardioversion shocks and magnesium sulfate [22]. An implantable cardioverter-defibrillator (ICD) should be considered if VA becomes intractable to pharmacological management [84], although it is not clear whether such action is indicated, particularly during hospitalization [21,22]. Indeed, one could resort to the use of a wearable ICD life vest in patients with recurrent VA during hospitalization, and consider implantation of an ICD based on monitoring during early follow-up, considering recovery of LV function, and while QTc is closely monitored [21,22].

### 3.17. Cardiac Arrest in Patients with TTS

Although cardiac arrest precipitated by VA or asystole is encountered in patients with suspected or established TTS [22], occasionally these arrhythmias are documented in patients presenting with out-of-hospital or in-hospital cardiac arrest, and found subsequently to have TTS. Thus, an amphidromic relationship between cardiac arrest and TTS may exist; accordingly, TTS with all its consequences may emerge in patients presenting with primarily potentially lethal VA and asystole [85,86], while also VA is a frequent complication of TTS. For more on the management of patients with TTS and cardiac arrest, Section 3.16. Considering the association of AF and VA and enhanced ASNS activity, it may be of value to monitor thoracic ECG signals indicative of stellate ganglia nerve input to the heart, via currently available ECG-based technology [60].

### 3.18. Pericarditis in Patients with TTS

Pericarditis (acute and chronic, including Dressler’s syndrome) with or without a pericardial effusion, and in association with or without cardiac tamponade has been described in patients with TTS; indeed, an amphidromic relation has been detected, in the sense that TTS may be complicated by pericarditis, and also TTS may be triggered by symptoms of pericarditis [87]. This association suggests caution in the use of thrombolytic therapy, anticoagulants, and glycoprotein IIb/IIIa inhibitors in patients with TTS and pericarditis [87]. Management is similar to idiopathic pericarditis, or pericarditis complicating other pathologies, and is based on the employment of ibuprofen or other non-steroidal anti-inflammatory drugs (NSAID), pericardiocentesis, and subxiphoid pericardial window [87].

### 3.19. Management of Comorbidities in Patients with Acute TTS

Management of present comorbidities in patients with TTS should continue while the patient has been admitted to the hospital and treated for TTS (Table 1 and Figure 1), providing that such therapy/ies does/do not precipitate clinical untoward responses to the patient. Accordingly, drugs for hypertension, HF, CAD, arrhythmias, diabetes, and hyperlipidemia should be continued, although doses in some of them can be modified to accommodate the new reality of TTS. Statins have been evaluated in patients with TTS, as summarized elsewhere [88], and they have not been found to exert any beneficial effects. Additionally, one should be cognizant of the occasional disease involvement of other organs (e.g., gastrointestinal tract, kidneys) precipitated by the pathophysiological processes that have triggered TTS [89]. In secondary TTS, when this disease is precipitated by physical triggers (e.g., sepsis, trauma, pulmonary insufficiency, diabetic ketoacidosis decompensation, endocrinological derangements, bleeding, surgery, anesthesia, neurologic/neurosurgical pathologies, and administration of chemotherapies), underlying precipitants should be appropriately managed, always keeping in mind that TTS is also present, and thus certain modification of provided therapies is in order. This applies primarily to the use of positive inotropes and vasodilator drugs which should not be employed in such patients with secondary TTS [22]. Instead phenylephrine for hypotension, and employment of ECMO and LVAD should be considered for severe HF and CS. In this context, detection of secondary TTS, triggered by any of the above pathologies [21], should require coronary angiography, which if it cannot be performed due to the gravity of the primary illness may lead physicians to resort to myocardial perfusion scintigraphy, or coronary computer tomography angiography (cCTA) [20] (although the latter two should not be considered equivalent to coronary angiography, which continues to be the “gold standard”); indeed, if none of the three can be performed, frequent employment of ECHO (including hand-held ECHO devices [90] in patients without a history or risk factors of CAD may be adequate for such differentiation, leaving only spontaneous coronary artery dissection as a TTS comorbidity requiring coronary arteriography [91]. Accordingly, the InterTAK group recommends cCTA as a reasonable option over coronary angiography for those with high InterTAK score and high pre-test probability for TTS; indeed, patients with low probability for TTS as per the InterTAK score, should undergo coronary angiography, while for patients with high such scores transthoracic ECHO may be considered adequate. [92]. The scenario of secondary TTS, triggered by a large array of acute medical and surgical illnesses, is exemplified by the frequent concurrent TTT and acute pathologies associated with hemodynamic instability, in patients cared for in intensive care units [93]. A great variety of pathologies triggering secondary TTS have specific management considerations, that should be taken into considerations, when one cares for such complex patient cases [21]. Indeed, a wider comorbid state has been implicated as the condition driving prognosis after TTS, particularly in connection with CS [94], prompting one to think that an intricate association of all existing comorbidities and the peculiarities of the pathophysiology of CS in TTS [20] contribute to the better recovery of LV function than seen in connection with CS in AMI, but to the worse prognosis during hospitalization and at follow-up. With this in mind, the importance of diagnosing and treating comorbidities is of great importance in this condition.

### 3.20. TTT Associated with CAD

Of note is that CAD and ACS, diseases that need to be differentiated from TTS, are occasionally comorbidities of TTS, and may occasionally precipitate secondarily TTS [95,96]. In such cases of concurrence of ACS and TTS, management of a coronary occlusion in a patient with AMI and TTS should be undertaken as performed routinely in accordance with issued guidelines for AMI, including the employment of percutaneous coronary interventions (PCI) [97], or even coronary artery bypass graft (CABG) surgery. In general, what is recommended is that complete revascularization be carried out particularly when myocardial ischemia/injury is due to left main coronary artery stenosis or severe multi-vessel CAD [20], along with all the preventive pharmacologic measures applied to patients with CAD and its manifestations during the acute phase of the disease and at follow-up.

## 4. Management of TTS in Potential Cardiac Donors

Cardiac transplantation is hampered by the scarcity of cardiac donors. This is further accentuated by the unsuitability of a proportion of donor heart grafts due to the development of secondary TTS in some of the potential cardiac donors [98]; it has been shown that heart grafts from heart donors who have suffered TTS with persisting or improving LV dysfunction have performed well without any adverse post-transplant outcomes [99]. Many of these potential heart donors with hearts revealing TTS features have suffered devastating neurological catastrophes due to head injuries or brain death from illicit drug overdoses. What is needed is to expedite the recovery of function in such heart grafts prior and after organ explantation, and during and after grafting to the hosts; these issues are currently being further investigated, and improvements need urgently to become systematized [100,101]. 

## 5. Current Follow-Up Management of Patients with TTS 

Management of patients who have been discharged after an episode of TTS aims at systematic attention at follow-up with an eye for monitoring for CAD risk factors, cardiovascular and other comorbidities, and recurrence of TTS (Section 6). Early on, follow-up should evaluate whether the LVWMAs have dissipated and the LVEF has returned to normal, or to a pre-TTS status level; this can be accomplished by a repeat ECHO, or a firstly performed cardiac magnetic resonance imaging (cMRI), which in addition to the assessment of LV function can provide insights about the resolution of LV thrombus, persistence of myocardial edema (ME), and presence of myocardial fibrosis and/or scarring [102]. The outcome of atrial arrhythmias and VA, which emerged during hospitalization, or appeared after discharge, and the associated QTc prolongation in the ECG, should be of utmost concern to the physicians. During the 1st and subsequent follow-up encounters, it should be ensured that all the complications noted during the hospitalization have been resolved or require attention (e.g., persisting arrhythmias or AV blocks requiring pharmacological treatment or implantation of cardiac electronic devices (PPM and/or ICD). A combination of ACEi/ARB led to decreased 1-year mortality, although this was not the case with administration of β-blockers [42]. Anticoagulation should continue for patients who had a thrombus while they were hospitalized, or in the rare occasions that LV function has not been fully restored. The treating physicians should investigate whether the patients continue to have symptoms, and whether such persisting morbidity could be attributed to TTS or other comorbidities, or the patients have returned completely to their health status preceding their TTS episode. Indeed, current evidence showing persistent structural, functional, and myocardial metabolic dysregulation at a follow-up of 13–39 months in patients with TTS [103], should prompt us to search for specific therapies (established and new) for managing the lingering morbidity of patients who have suffered TTS. 

## 6. Current Therapy Aimed at Preventing Recurrence of TTS

Therapy for recurrence of TTS should include regular follow-up aiming at monitoring for and managing of risk factors for CAD and other comorbidities, favoring the use of ACEi/ARB and long-acting β-blockers (e.g., metoprolol, bisoprolol) needed for patients with comorbidities, but even for patients without such comorbidities. 

However, β-blockers, ACEi/ARB, and aspirin have not prevented recurrence of TTS, its severity, or led to better survival [21,22,92,104,105], while a meta-regression study of the combined use of β-blockers and ACEi/ARB revealed a lower recurrence rate of TTC [106], a finding that requires replication. Additionally, aspirin, a frequently administered drug in patients with or suspected atherosclerosis, following discharge of patients with TTS, has not influenced favorably outcome at follow-up [107]. Endocrinological comorbidities (e.g., thyroid disorders, pheochromocytoma) should be sought after in an effort to fend off recurrence of TTS. Pheochromocytomas/paragangliomas in particular have often repeatedly been missed, and are diagnosed after recurrent episodes of TTS [108]. Considering the high rate of neurological and psychiatric comorbidities, and substance abuse in patients with TTS [42,109,110,111], physicians following patients with an index episode of TTS should consider systematic longitudinal management of such neurological/psychiatric pathologies, which may influence the rate of TTS recurrence. Of particular importance herein is the initiation or uptitration/downtitration of psychotropic drugs in the management of epilepsy, depression, and anxiety and the recurrence of TTS [111,112]; thus, such changes in the drug doses should be carried out in close collaboration of the patient, cardiologist, neurologist, and psychiatrist, who should also provide psychological counseling, psychotherapy, including cognitive behavioral therapy [21,22]. Currently there is no evidence that such therapeutic considerations have prevented recurrence of TTS. Additionally, expert management of chronic pulmonary pathology is recommended [22], since often decompensated chronic obstructive lung disease and asthma serve as triggers for the emergence of TTS, perhaps primed by the excessive use of bronchodilators (β_2_-agonists). Since malignancies and TTS are intricately associated [113], the treating physician should look for yet undiagnosed underlying malignancy in patients with past history of TTS, particularly in the absence of obvious trigger(s) [21]; also, in patients with malignancies as a comorbidity to TTS, we should be proactive in helping our patients undergoing diagnostic procedures and pharmacological, surgical, and radiation therapies, since such exigencies are associated with recurrent TTS. Indeed, such an approach should be generalized to all patients with previous history of TTS, and patients should undergo procedures/surgeries after pretreatment over a course of a few days to weeks, with long-acting β-blockers, or as an alternative be supported with periprocedural/perioperative continuous infusions of short- or ultrashort-acting β-blockers (e.g., esmolol or landiolol). The efficacy of this hypothetical preventive approach for TTS recurrence has not been shown, but its plausible merit needs to be explored. Considering the higher propensity of women to suffer TTS, and animal models showing a preventive role of estrogens in the emergence of TTS [114], there are no clinical data supporting use of estrogens either to prevent index TTS episodes or their recurrence; however, it may be of value to reexplore the issue of a low dose estrogen supplementation for perimenopausal and postmenopausal women with the intention to prevent TTS or its recurrence [21,115].

## 7. Future Therapeutic Options for TTS

Considering the delays in making the diagnosis of TTS due to its similarity in clinical presentation with CAD, ACS, AMI, and HF, and the hard fact that the victims of TTS, before and after the diagnosis has been suspected and eventually established, are treated for a number of hours and often few days with therapies designed and proven beneficial for other cardiovascular pathologies, it may be unlikely that RCT could be carried out to explore for therapies which will make a difference in the management of patients with TTS. However, it is conceivable that “specific for TTS therapies” may emerge in the future, mediating an amelioration or complete reversion of pathophysiologic mechanisms, which have caused the TTS phenotype. Such therapies, may or may not be efficacious in improving the clinical course of patients with TTS, accelerating the healing process, preventing major cardiovascular and non-cardiovascular complications, and shortening the hospital stay. An impediment for a therapy that would be decisively beneficial in the management of TTS is that, even if such a therapy becomes available, it could only be implemented after the TTS is already established and the morbid pathology with its consequences is in the process of recovery. Although we do not know whether an autonomic central nervous system catecholamine storm with resultant local overstimulation of cardiomyocytes, a surge in the blood-borne catecholamine levels, spasm of the epicardial coronaries and/or coronary microcirculation, endothelial dysfunction, negative myocardial supply/demand ratio, developed LVOTO, or some other(s) not suspected as yet pathomechanism(s) are at work, what clinicians or researchers are presented with is a probably transiently dysfunctional myocardial territory, not unlike the stunned myocardium, with the features of reperfusion injury, encountered in ACS and AMI [116]. 

A hypothetical example of the dissociation between the pathophysiological process leading up to TTS, and a therapy designed to reverse such a nosogenic scenario can be visualized by the following: let us assume that TTS is caused by an intense relatively prolonged (more than what is encountered in Prinzmetal’s angina) coronary vasospasm, affecting the epicardial coronaries and/or the coronary microcirculation, and resulting in a region with features of stunned myocardium and reperfusion injury [116]; employment of nitroglycerin, organic nitrates, or calcium blockers are not expected to exert a significant therapeutic effect leading to recovery of the myocardial region affected by the ischemic/reperfusion injury, since coronary vasospasm is not anymore exerted, or even if it is, the resultant myocardial damage has been already completed; thus even therapy along reversing the pathophysiologic trajectory resulting in TTS, does not constitute an effective management approach for established TTS. 

**Figure 7:**
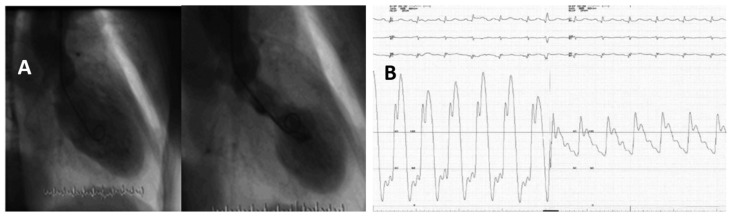
A patient with TTS, LVOTO, and MR treated with the ultrashort-acting β-blocker landiolol. A 72-year-old woman presented with chest pain and dyspnea triggered by emotional stress and was diagnosed with TTS and LVOTO. Her HR was 100 beats/min, BP was 96/60 mmHg. and a systolic murmur in the second right sternal border was heard. Coronary angiography did not disclose significant lesions, but LV angiography showed an apical variant TTS (**A**), MR due to anterior mitral valve leaflet SAM, with a pull-back from the LV apex to basal tract of the LV, revealing a peak gradient of 61.9 mmHg (**B**), suggestive of LVOTO. Infusion of landiolol at a dose of 4 μg/kg/min was started, which was uptitrated to 6, 8, and finally 10 μg/kg/min, under close monitoring of HR, BP, and ECHO, at which point the BP increased to 120 mmHg, the HR dropped to 50-60 beats/min, the LV gradient decreased to 20 mmHg, and the MR became mild. The dose of landiolol was decreased to 4 μg/kg/min on day 3, and the drug was terminated on day 4. Subsequently her course was uneventful and she was discharged on day 10. Reproduced and modified from Ref. [38], with the permission of Journal of General and Family Medicine.

**Figure 8:**
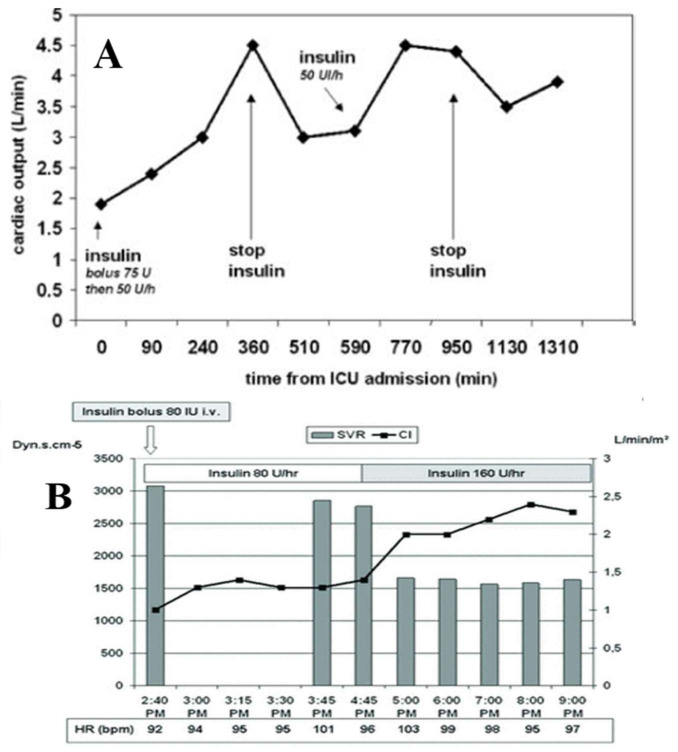

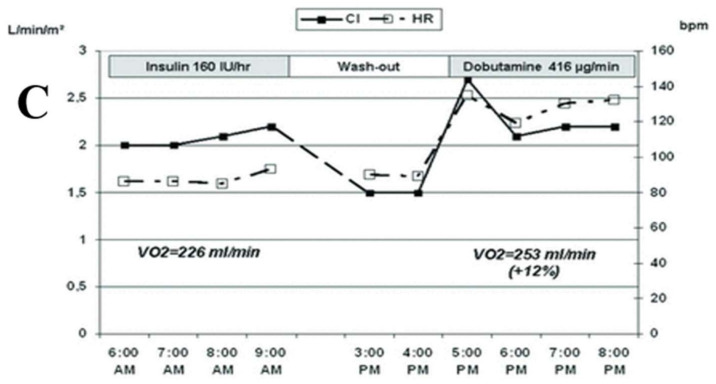
Hemodynamic changes in two patients with TTS treated with high doses of insulin. Effects of insulin in two patients, one with TTS after subarachnoid hemorrhage (**A**), and the other with TTS after a stroke (**B**,**C**). Vide response of cardiac output to the infusion and subsequent withdrawal of insulin (**A**). Vide response of cardiac index and SVR to 2 doses of insulin, and the stability of HR (**B**). Vide the advantages of the response of cardiac index, HR, and VO_2_ to insulin versus dobutamine (**C**). Reproduced and modified from Refs. [119,121], with the permission of the Neurosurgery and BMJ Case Report.

It is conceivable that a diverse variety of pathophysiologic entities (ACS, AMI, Prinzmetal’s angina, and TTS) could lead to the *same* pathophysiologic/pathoanatomic/pathohistologic outcome, i.e., that of “ischemic/reperfusion injury” [4], and thus therapies previously proposed for ACS and AMI [8,9,116], deserve a trial in patients with TTS [10,11]. Accordingly, it has been recently proposed that large doses of insulin infusions, in connection with careful monitoring to prevent hypoglycemia and hypokalemia, via concomitant infusions of dextrose and potassium supplementation, in conjunction with intravenous use of short-, or ultrashort-acting β-blockers (e.g., esmolol or landiolol) [39] (Figure 7) perhaps have beneficial therapeutic effects in patients with TTS [10,11], addressing specifically the devastating metabolic impairment (glucose and lipid pathways dysregulation, leading to decreased final glycolytic and β-oxydation metabolites and reduced availability of Krebs intermediates), noted in TTS [117,118]. This proposal is based on previous literature of animal models of stress cardiomyopathy, recently summarized [10], and limited experience in patients with neurogenic cardiomyopathy and TTS [37,38,119,120,121,122] (Figure 8). Consequently. a trial including the above therapeutic scheme deserves consideration in the management of patients with TTS. Justification of insulin employment in TTS is supported by studies using ^18^F-FDG uptake confirming the presence of glucose metabolism disorder, similar to that observed in stunned or hibernated myocardium [123], for which insulin has been proposed [9].

An additional therapeutic parallel, tangentially supporting the consideration of insulin in the management of TTS, is the recommendation of incrementally administered high dose insulin therapy, along with intravenous calcium, in patients with drug-induced cardiac toxicity engendered by calcium blockers [124]. Additionally, since there are previous recommendations for implementation of intravenous lipid-emulsion therapy in patients with ischemic stunned myocardium/reperfusion injury [124], and calcium-blockers or β-blockers overdose [125], such therapy could be tested in animal TTS models, and if found safe and useful, be further evaluated for patients with TTS. However, we should always exercise restraint in the notion that what we view in TTS represents an adaptive protective response to the autonomic adrenergic overstimulation of the heart, and thus we should be concerned by interfering in the spontaneous self-healing process [1,20,21]. 

Since experiments in a TTS rodent model with catecholamine-triggered TTS revealed that isoflurane anesthesia exerted a protective effect for development of LV dysfunction [126,127], it would be of great interest to evaluate even in a limited cohort this therapy’s impact in human TTS. 

Another matter of concern is the continuation of ill health at follow-up of patients with TTS with evidence of inadequate recovery of contractile function, metabolic dysfunction and systemic inflammation several months after the TTS episode [103,128,129]. What therapy should be implemented to manage these persisting problems is not currently evident. Among other well-established therapies one could consider continuation of therapy with insulin, proposed for the acute phase of TTS (vide supra), which may provide benefits to such patients. Accordingly, a time period with low doses of insulin as for diabetics with enhanced oral intake of carbohydrates and potassium to avoid hypoglycemia and/or hypokalemia, may be worth of preclinical and clinical exploration, for its effects on the post-TTS lingering morbidity.

In reference to the underlying systemic inflammation, one wonders whether a 4-pronged RCT, assigning patients to placebo, NSAID, colchicine, and corticosteroids, with an evaluation at 6 and 12 months post the episode of TTS, and assessment of symptoms, cardiopulmonary stress test, brain natriuretic peptides, C-reactive protein, ECHO strain imaging, and cMRI, may provide some answers.

A flood of articles is currently being published pertaining to the association of TTS in patients with COVID-19 admitted to intensive care units [130]. It cannot be overemphasized that a high index of suspicion needs to be imparted to all physicians that TTS may occur, and probably remains underdiagnosed, in patients with COVID-19. Concerns of the care givers should be geared towards the detection and prompt response to HF, CS, drug-induced prolongation of the QTc, and life-threatening VA in patients with COVID-19 and complicated secondary TTS [128] Finally, the heavy reliance on the frequent implementation of ECHO in patients admitted with COVID-19 cannot be overemphasized [90].

## 8. Prognosis of TTS

TTS, although initially thought to be an entirely benign condition, is currently felt to be a serious illness, with rates of morbidity and mortality comparable to those experienced after an AMI or other ACS [1,2,20,21]. In addition, a lingering morbidity, characterized by exercise intolerance, atypical for ischemia chest pain, exertional dyspnea, and metabolic abnormalities, persist for many months after an index episode of TTS [103]. Some patients suffer recurrence of TTS, with rare some developing multiple such episodes [131]. The early detected LV dysfunction thought initially to fully recover in a few weeks to months, based on conventional transthoracic ECHO, has been found not to be fully restored to normalcy by strain ECHO deformation studies, and some interstitial fibrosis is documented by cMRI to replace the initially observed ME [132]. In addition, patients with TTS have a high rate of cancer, neurological and psychiatric diseases [133,134], and common variety comorbidities (i.e., hypertension, diabetes), sometimes to a greater degree than patients with CAD/AMI/ACS [135], resulting to an even higher morbidity/mortality phenotypes than the latter. The issue of the prognostic role of diabetes is still unsettled, considering that a “diabetes paradox” has been identified exerting a protecting influence in the emergence of TTS, and resulting in amelioration of complications during hospitalization [136]. In general, a large array of symptoms/signs, laboratory findings, complications, comorbidities, and prognostic scores have been demonstrated, in small and large TTS patient cohorts, to be associated with worse outcome (both short-term and long term), with Table 2 providing a non-all-inclusive list of such predictors. Inconsistencies regarding prognosticators among studies, are probably related to variation in the composition of examined cohorts and/or their sizes. Finally, it appears that there are no predictors from the index episode of TTS of subsequent TTS recurrence(s) [137], and that ACE1/ARB, but not β-blockers, instituted at discharge following a TTS episode, may prevent recurrence [21,138], although this is not firmly established [42,79,88]. 

## 9. Conclusions

The pathophysiology of TTS continues to be elusive and its management is currently based on the extrapolation of therapeutic practices proven/established for other cardiovascular diseases (CAD, AMI, HF, etc.). This may not be totally inappropriate since a secure diagnosis of TTS is made always with significant delays and thus by necessity supportive therapies in response to common symptoms and signs of cardiovascular pathology are necessary. Additionally, we should entertain the notion that whatever is the pathoetiology of TTS, the eventual emergence of stunned/reperfusion myocardial injury may call for management approaches not unique only to TTS. Due to eventual spontaneous reversibility of such pathology, frequently noted in patients with TTS, observing and monitoring asymptomatic or mildly symptomatic patients with adequate organ perfusion, while self-healing takes place may be advisable along the principle of “first do no harm”. Continuation of therapies for previously present comorbidities should not be interrupted with uptitration/downtitration of drugs, as necessary. ACEi/ARB, short-acting β-blockers, anticoagulants are often implemented during hospitalization, and continued during follow-up, as needed. Earlier employment of ECMO and/or LVAD may be needed for patients in CS, and monitoring for the emergence of lethal VA, particularly associated with prolongation of the QTc interval, is imperative. Consideration should be given to favoring short- and ultrashort-acting β-blockers and implementation of large doses of insulin in the management of patients with TTS and HF or CS. Finally, the management of patients who have suffered TTS should be systematized at follow-up to include use of ACEi/ARB, reexamination of whether β-blockers prevent recurrence, employment of psychiatric evaluation and therapy, reevaluation of the inciting role of malignancy in the emergence of recurrent TTS, and management of comorbidities as they relate to recurrence of TTS.

## Figures and Tables

**Table 1:** Complications of TTS and corresponding recommended therapies.

Complications	Recommended Therapy (ies)
**Reevaluate frequently!**	**Adjust accordingly**
Asymptomatic; negative physical exam	Observe; supportive care
Angina	Nitrates; optimize volume short/ultrashort-acting β-blockers
Hypertension	Continue previous: antihypertensive regimen short/ultrashort-acting β-blockers
Tachycardia	Short/ultrashort-acting β-blockers
Hypotension	Optimize volume; D/C β-blockers; R/O LVOTO CS; phenylephrine
Bradycardia; AV blocks	D/C β-blockers; atropine pacemaker
Dyspnea; pulmonary congestion	Diuretics; oxygen
Heart failure	Diuretics; oxygen ACEi/ARB; levosimendan
Cardiogenic shock	Levosimendan; ECMO LVAD
Prolonged QTc	D/C β-blockers Pacemakers; monitoring
Atrial arrhythmias other than atrial fibrillation	β-blockers; monitoring
Atrial fibrillation	heparin; LMWH; vitamin K antagonists
PVCs/NVT	β-blockers; monitoring
Sustained VT	DC cardioversion
Ventricular fibrillation	DC-cardioversion
Ischemic stroke	Heparin; LMWH; vitamin K antagonists
Systemic or pulmonary embolism	Heparin; LMWH; vitamin K
LV thrombus	Heparin; LMWH; vitamin K antagonists
Ischemic stroke with LV thrombus	Heparin; LMWH; vitamin K antagonists; consult with Neurology
Hemorrhagic stroke with LV thrombus	Consult with Neurology and Cardiothoracic Surgery
Low LVEF with large apical akinesis/dyskinesis	Consider heparin; LMWH vitamin K antagonists
Mitral regurgitation	Optimize volume; diuretics R/O LVOTO
LVOTO	β-blockers; phenylephrine
LVOTO with CS	β-blockers cautiously vibradine; pacemaker
Left ventricular rupture	Stop anticoagulation; consult with Cardiothoracic Surgery
Right ventricular involvement	Monitor closely; diuretics
Pericarditis	Frequent ECHOs; NSAIDs consider stopping anticoagulation therapy
Torsades de pointes	Stop β-blockers; monitor QTc; pacemaker
Comorbidities	Manage as done routinely with modification as needed
Prior prescribed drugs	Continue/stop/modify as needed
Acute kidney injury	Monitor renal function optimize volume; consider hemodialysis
Associated AMI/ACS	Manage as needed including revascularization
Associated SCAD	Manage as needed including revascularization
Cardiac arrest	Resuscitation; consideration for vest and/or ICD
Anxiety/depression	Consult with Psychiatry
**Reevaluate frequently!**	**Adjust accordingly!**

Abbreviations: ACEi/ARB = angiotensin-converting enzyme inhibitors/angiotensin receptor blockers; AMI/ACS = myocardial infarction/acute coronary syndromes; AV = atrioventricular; CS = cardiogenic shock; D/C = discontinue; ECHO = transthoracic echocardiogram; ECMO = extracorporeal membrane oxygenator; ICD = implanted cardioverter-defibrillator; LMWH = low-molecular-weight heparins; LV = left ventricle; LVAD = left ventricular assist device; LVEF = left ventricular ejection fraction; LVOTO = left ventricular outflow tract obstruction; NSAIDs = non-steroidal anti-inflammatory drugs; NVT = non-sustained ventricular tachycardia; PVCs = premature ventricular complexes; R/O = rule out; SCAD = spontaneous coronary artery dissection; VT = ventricular tachycardia.

**Table 2:** Symptoms/signs, laboratory findings, complications, and comorbidities of patients with TTS, associated with worse prognosis.

Symptoms/Signs	Laboratory Findings	Complications	Comorbidities	Prognostic Scores
Tachycardia Hypotension High respiratory rate High temperature Persisting angina Dyspnea Age >70 Age <50 Male sex Physical stressors	High sensitivity troponin Hyperglycemia Hypoxia High brain natriuretic peptides Long QTc LVEF <30% High E/e’ Low LV-GLS T-wave inversion LV concentric hypertrophy Sigmoid septum ST-segment elevation Marked LVWMAs Apical variant Atypical ballooning High blood norepinephrine High tumor necrosis factor-α Myocardial edema Late recovery of LVEF High C-reactive protein High WBC count- Anemia Δnegative T-wave amplitude dispersion ΔQT dispersion; Low T3 TTS right ventricular involvement Low BMI Low eGFR cMRI-detected fibrosis Thrombolysis in myocardial infarction-2 flow cMRI late gadolinium enhancement	HF CS VA Cardiac arrest Asystole Pulseless electrical activity LV dysfunction requiring MCS Stroke Acute renal failure AF Respiratory distress needing mechanical ventilation Pulmonary edema Need for catecholamine use Need for inotropic drugs Killip class III/IV Asystole CHB MR Intraventricular thrombus	Diabetes Hypertension Neurological pathologies Psychiatric diseases Malignancies Acute pulmonary triggers Endothelial dysfunction Secondary TTS CAD Trauma Need for resuscitation eGFR Multiple noncardiac comorbidities Sepsis Admission to the ICU Peripheral artery diseas Chronic renal failure	Killip class (III and IV) on admission High GRACE score InterTAK Classification InterTAK Prognostic Score CHA2DS2-VASc risk score German and Italian Stress Cardiomyopathy (GEIST) Score

Abbreviations: AF = atrial fibrillation; BMI = body mass index; CAD = coronary artery disease; CHB = complete heart block; cMRI = cardiac magnetic resonance imaging; CS = cardiogenic shock; Δ = change; e GFR = estimated glomerular filtration rate; HF = heart failure; GLS = global longitudinal strain; ICU = Intensive Care Unit; LV = left ventricular; LVEF = left ventricular ejection fraction; LVOTO = left ventricular outflow tract obstruction; LVWMAs = left ventricular wall motion abnormalities; MCS = mechanical circulatory support; TTS = takotsubo syndrome; VA = ventricular arrhythmias; WBC = white blood cells.

## Data Availability

Not applicable.

## Abbreviations

ACS	Acute Coronary Syndromes
ACEi	Angiotensin-Converting Enzyme Inhibitors
AF	Atrial Fibrillation
AMI	Acute Myocardial Infraction
ARB	Angiotensin Receptor Blockers
AV	Atrioventricular
BP	Blood Pressure
CABG	Coronary Artery Bypass Graft
CAD	Coronary Artery Disease
CHB	Complete Heart Block
cCTA	Coronary Computed Tomography Angiography
cMRI	Cardiac Magnetic Resonance Imaging
CS	Cardiogenic Shock
ECG	Electrocardiogram (phic)
ECHO	Transthoracic Echocardiography (gram)
ECMO	Extracorporeal Membrane Oxygenator
HF	Heart Failure
HR	Heart Rate
IABP	Intra-aortic Balloon Pump
ICD	Implantable Cardioverter-Defibrillator
LV	Left Ventricle (cular)
LVEF	Left ventricular Ejection Fraction
LVAD	Left Ventricular Assist Device
LVWMAs	Left Ventricular Wall Motion Abnormalities
LMWH	Low-Molecular-Wight Heparins
LVOTO	Left Ventricular Outflow Tract Obstruction
MCS	Mechanical Circulatory Supprot
ME	Myocardial Edema
MR	Mitral Regurgitation
NSAID	Non-steroidal Anti-inflammatory Drugs
PCI	Percutaneous Coronary Artery Intervention
PPM	Permanent Pacemaker
RCT	Randomized Controlled Trials
RV	Right Ventricle (cular)
SAM	Systolic Anterior Motion
SVR	Systemic Vascular Resistance
TdP	Torsades de Pointes
TTS	Takotsubo Syndrome
VA	Ventricular Arrhythmias
VT	Ventricular Tachycardia
